# Raman Spectroscopy and Spectral Signatures of AlScN/Al_2_O_3_

**DOI:** 10.3390/mi13111961

**Published:** 2022-11-11

**Authors:** Dmytro Solonenko, Agnė Žukauskaitė, Julian Pilz, Mohssen Moridi, Sarah Risquez

**Affiliations:** 1 Microsystems Division, Silicon Austria Labs, 9524 Villach, Austria; 2 Fraunhofer Institute for Applied Solid State Physics, IAF, Tullastr. 72, D-79108 Freiburg, Germany

**Keywords:** aluminium scandium nitride, piezoelectric films, Raman spectroscopy, alloy scattering, temperature coefficient

## Abstract

III-V solid solutions are sensitive to growth conditions due to their stochastic nature. The highly crystalline thin films require a profound understanding of the material properties and reliable means of their determination. In this work, we have investigated the Raman spectral fingerprint of ${\mathrm{Al}}_{1-x}$${\mathrm{Sc}}_{x}$N thin films with Sc concentrations *x* = 0, 0.14, 0.17, 0.23, 0.32, and 0.41, grown on ${\mathrm{Al}}_{2}$$O_{3}$(0001) substrates. The spectra show softening and broadening of the modes related to the dominant wurtzite phase with increasing Sc content, in agreement with the corresponding XRD results. We investigated the primary scattering mechanism responsible for the immense modes’ linewidths by comparing the average grain sizes to the phonon correlation length, indicating that alloying augments the point defect density. The low-frequency Raman bands were attributed to the confined spherical acoustic modes in the co-forming ScN nanoparticles. Temperature-dependent Raman measurements enabled the temperature coefficient of the $E_{2}$(high) mode to be determined for all Sc concentrations for the precise temperature monitoring in AlScN-based devices.

## 1. Introduction

Aluminium nitride (AlN) thin films, being a staple for commercial acoustic wave resonators, have been well investigated over the last two decades to improve understanding of the growth–performance relation [1,2,3]. The advent of the ${\mathrm{Al}}_{1-x}$${\mathrm{Sc}}_{x}$N (AlScN) pseudobinary alloys [4] instigated further studies on the enhancement of the piezoelectric properties, yielding the interplay of the wurtzite and hexagonal lattice configurations, which led to the overall lattice softening [5] and the increased electromagnetic coupling [6]. The phase formation diagram is complicated by the fact that pure ScN crystallises in the rock-salt structure [7]. It was initially shown that only up to *x* = 0.22 could be introduced into the AlN lattice before it converts to the cubic system [8]. However, the maximum amount of Sc within the wurtzite lattice was later optimised: *x* = 0.41 [9]. The amount of Sc atoms dispersed in the AlN wurtzite lattice has a dominant role on the physical properties of the AlScN, which can, for instance, show even ferroelectric behaviour for *x* > 0.28 [10].

The phonon properties of these pseudobinary alloys are of great interest not only to enable insights into the structural integrity and thermal properties of their crystals, but also to provide reliable and non-destructive characterisation of the thin films in terms of their dielectric and optical properties. The first results on the infrared-active phonon modes were shown by Mayrhofer et al. for AlScN films of varied Sc composition in a narrow range up to 15% [11]. The redshift of the $E_{1}$(TO) band of AlN was shown to be proportional to the Sc content, which was interpreted as the indication of the elongating M-N (M = Al, Sc) bonds. Similar behaviour was also observed for the $E_{2}$(high) and $A_{1}$(TO) Raman bands and compared to the evolution of the rest IR-active bands, thereby confirming a decrease in the bond length as a result of increased bond ionicity [12].

Despite the narrow range of the Sc concentration, the main trends in phonon properties of the AlScN lattice have been unravelled so far while largely omitting the obvious impact of the film’s microstructure on the spectral data. While the phonon frequency shift is evident and expected in the framework of the lattice softening, the rest of the peak characteristics are intricately entangled with the quality of the AlScN films. Although the influence of the microstructure can be observed using both IR and Raman spectra, it can be approached more accurately in the latter case [13,14]. Indeed, the large peak broadening observed in the Raman spectra of AlScN alloys remains poorly understood, and so does their overall spectral signature, which includes a few Raman bands addressed earlier [12,15,16,17,18,19]. It is necessary for understanding the contributions of various mechanisms responsible for the dramatic broadening of the Raman-active spectral bands, such as the possibility of the rock-salt phase formation, alloy- or disorder-based phonon scattering, and size effect.

In this work, we investigated in detail the vibrational signatures of ${\mathrm{Al}}_{1-x}$${\mathrm{Sc}}_{x}$N pseudobinary alloys in a broad range of the Sc concentrations (up to *x* = 0.41). Our results confirm the previous observations of the frequency shift and peak broadening for the Raman bands observed in the c–axis–oriented films [12,15]. We show that the Raman spectra of the alloys actually exhibit up to eight bands. Unreported bands are attributed to the second-order phonon modes of the wurtzite AlScN lattice. Moreover, we investigated the low–frequency spectral region, the investigation of which is usually limited by technical difficulties. We found the presence of Raman bands which are proposed to be related to the confined acoustic modes. In contrast to pure AlN, the second–order spectral features stemming from two-phonon modes and phonon density of states are greatly enhanced as a result of the bandgap shrinkage and a high density of the midgap states. The presence of these electronic states is attributed to the point defects, the density of which is two orders of magnitude higher than in pure wurtzite, as assessed via the phonon correlation length. This was corroborated by temperature–dependent Raman measurements showing that the phonon-defect scattering dominates over the phonon–phonon mechanism in AlScN regardless of the Sc concentration. The determined temperature coefficients can be used for the precise temperature determination in AlScN films via Raman spectroscopy.

## 2. Materials and Methods

First 1 μm thick ${\mathrm{Al}}_{1-x}$${\mathrm{Sc}}_{x}$N layers with Sc concentrations *x* = 0, 0.14, 0.17, 0.23, 0.32, and 0.41 were deposited on single-sided, polished, 100 mm diameter ${\mathrm{Al}}_{2}$$O_{3}$(0001) substrates at chuck temperatures of 300–400 °C by reactive pulsed DC magnetron co-sputtering (Evatec cluster sputter tool). In addition 99.9995% pure Al and 99.99% pure Sc targets were sputtered in a pure $N_{2}$ atmosphere; a constant total power applied to the Al and Sc targets to achieve different Sc concentrations; all other growth parameters were kept constant. The deposition parameters are described in detail in [9,20]. The Sc content with an accuracy of x ± 0.02 was determined by energy dispersive X-ray (EDX) spectroscopy (Bruker Quantax, Bruker Corporation, Billerica, MA, USA) in a scanning electron microscope (Zeiss Auriga Crossbeam FIB-SEM, Carl Zeiss, Oberkochen, Germany). The compositional analysis of the samples is described elsewhere [21]. The θ/2θ scans were obtained using a X-ray diffractometer equipped with a 4-bounce Ge 220 monochromator, a parallel beam X-ray mirror on the incident side, and a PiXcel3D detector (X’${\mathrm{Pert}}^{3}$ MRD XL, PANalytical, Almelo, The Netherlands). An atomic force microscope (NX20, Park Systems, Suwon, Korea) with the AC160TS tip (radius: 2 nm) was used to investigate the surface morphology and determine the roughness using Gwyddion software [22]. Raman spectra were collected using the micro-Raman spectrometer (inVia Qontor, Renishaw, London, UK). The excitation wavelength of the DPSS laser (Renishaw RL532) was 532 nm when used in combination with the 2400 L/mm grating, yielding the spectral resolution of about 0.1 ${\mathrm{cm}}^{-1}$. The power density of about 10 mW$\cdot \mu m^{-2}$ estimated for the 100× objective (NA = 0.75) was used to avoid the heating of samples. The near-excitation tunable (NExT, Renishaw) filter was used, enabling the collection of the anti-Stokes spectra. The baseline stemming from the photoluminescence was subtracted from all obtained spectra using a polynomial function, describing the background signal increasing towards longer wavelengths. The temperature-dependent Raman measurements were recorded using a thermostat, a hot plate-like stage (T96-P, Linkam Scientific Instruments, Tadworth, UK). Due to the heating of the samples from backside of the sapphire substrate, it was important to realise that the heating of the AlScN films corresponded to the temperature setpoints. Provided the outstanding thermal conductivity of sapphire, the temperature in the films was assumed to be equal to that of the sapphire substrate. The calibration and tracking of the sample’s temperature was performed using the $A_{1g}$ Raman-active mode of the sapphire substrate (417.4 ${\mathrm{cm}}^{-1}$ [23]). The thermal coefficient of the Raman band was found to be −0.015 K·${\mathrm{cm}}^{-1}$, which is in an excellent agreement with previous studies [24].

## 3. Results and Discussion

### 3.1. Film Microstructure

**Figure 1:**
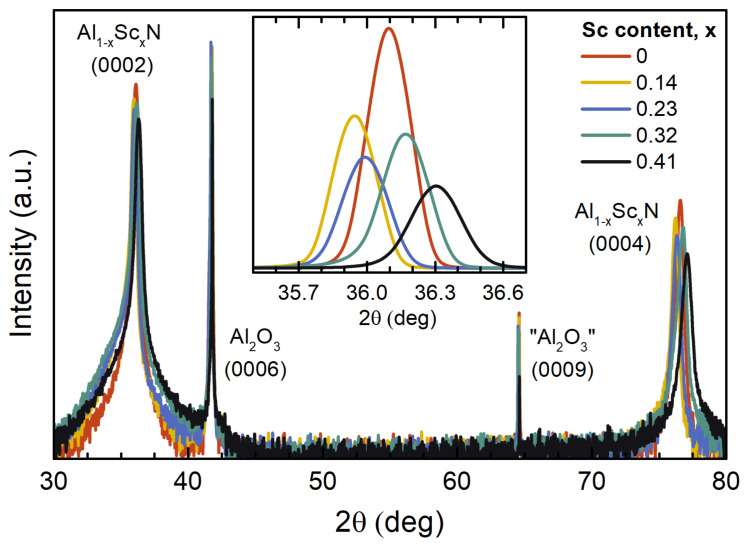
XRD symmetric θ/2θ scans of AlScN films with various Sc amounts. Inset: the 2θ range in the vicinity of the (0002) peak of the AlScN alloy.

The XRD scans of the ${\mathrm{Al}}_{1-x}$${\mathrm{Sc}}_{x}$N (*x* = 0, 0.14, 0.23, 0.32, 0.41) films grown on the c-plane ${\mathrm{Al}}_{2}$$O_{3}$ substrates show the 000*l* (*l* = 2, 4) reflections of the nitride and 000*l* (*l* = 6, 9) reflections of the oxide compounds (Figure 1). The lack of additional peaks assigned to the AlScN and the reflections stemming from the c-plane suggest that the films are highly c-axis-oriented. Pole figure measurements confirm in-plane oriented growth of AlScN with the epitaxial relationship defined as [10-10]AlScN//[11-20]sapphire and (0001)AlScN//(0001)sapphire [21]. Provided that the film thickness values are similar, the variation in the peak intensity indicates different amounts of the diffracting domains, and the variation in the peak linewidths suggests their diverging size distributions (Figure 1, inset). The trends for the peak position, which are dependent on the Sc concentration and the thermal strain, are in agreement with the previously reported ones [20,25,26]. The alterations in the peak positions and linewidths of the (0004) reflection peak mirror the behaviour observed for the (0002) one scaled due to the higher 2θ angles, which suggests good uniformity for the long-range order. The peak intensity is continuously reduced for higher amounts of Sc, which can be related to the size reduction of crystalline domains. No peak solely related to the rock-salt ScN or AlScN phase, which might have been expected in the alloy phase diagram [8], was observed in the diffractograms, confirming the dominating wurtzite phase in the pseudobinary AlScN alloy.

**Figure 2:**
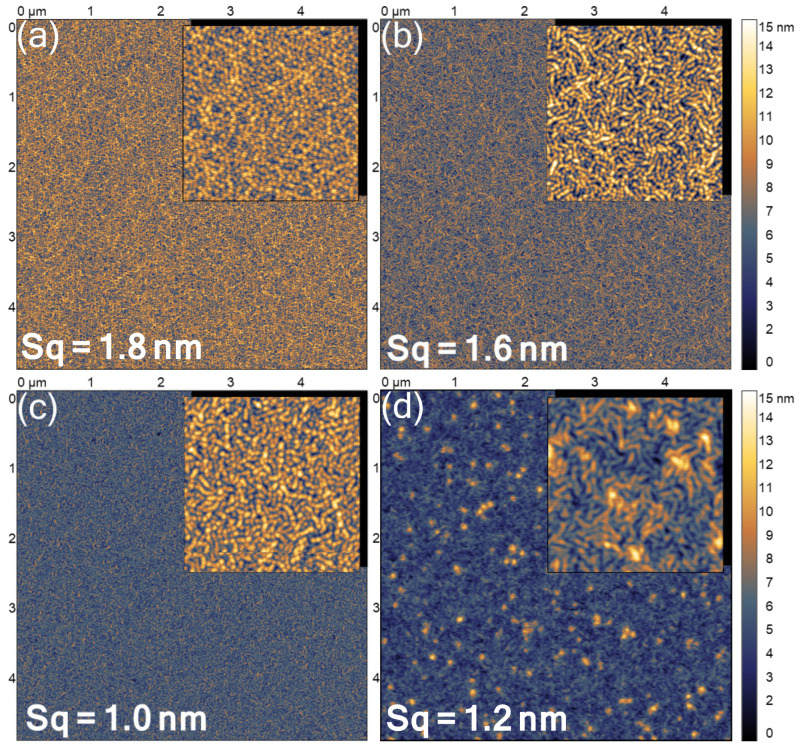
Surface morphology of the thin films with the following Sc content, *x* = (**a**) 0 (AlN), (**b**) 0.14, (**c**) 0.32, (**d**) 0.41 obtained via AFM. The sample with *x* = 0.23 is not shown. The false colour scale is common for all images. The rms roughness, *Sq*, values is indicated on the corresponding images. Insets: complimentary 1×1 $\mu m^{2}$ AFM images.

Figure 2 shows AFM micrographs of the films, revealing the pebble-like morphology characteristic of the AlN films grown via magnetron sputtering [27,28]. The AFM results revealed a less random arrangement of the grains being seemingly clustered into short chains of hemispherical droplets, which resembled the surface of AlN after the annealing process [29] and thus indicated the relation to the alteration of the adatom surface mobility with adding more Sc. When applying the structure zone model [30], the worm-like surface texture is characteristic for the low adatom mobility [31], which is caused by the different kinetic energies of co-sputtered species. The surface of the ${\mathrm{Al}}_{0.59}$${\mathrm{Sc}}_{0.41}$N film reveals the presence of larger grains located at the nodes of the worm-like surface. These specific grains may be attributed to the abnormally oriented grains of AlScN observed for the cases of high Sc concentration [21,32]. The surface roughness of the films decreased when alloying to more Sc atoms and reached its minimum value for *x* = 0.32 of the scandium composition. The following increase in the roughness values for the film alloyed with the highest Sc amount was related to the bright protrusions visible in the image. Nevertheless, the roughness values below 2 nm revealed that AlScN films were largely smooth and exhibited no surface structures to be assigned to other crystalline phases. Thus, the investigation of the film microstructure showed that the films of the pseudobinary AlScN alloys on ${\mathrm{Al}}_{2}$$O_{3}$ consisted of one crystalline wurtzite phase, which was oriented along the c-axis, indicating columnar growth.

### 3.2. Vibrational Properties

**Figure 3:**
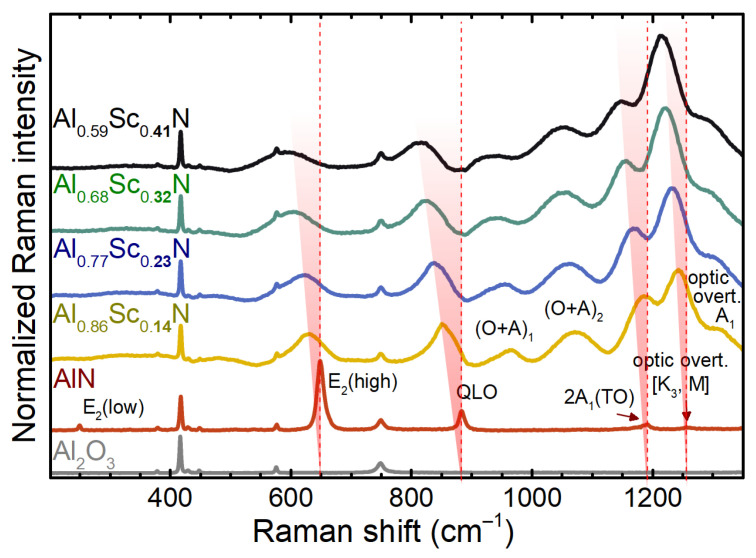
Raman spectra of AlScN films with various Sc concentrations. The spectra are stacked for clarity. The spectral bands of pure AlN are labelled by the dash lines to emphasise their low-frequency shifts in ${\mathrm{Al}}_{1-x}$${\mathrm{Sc}}_{x}$N as a guide to the eye. The Raman spectrum of the ${\mathrm{Al}}_{2}$$O_{3}$ substrate revealed the bands unrelated to the nitride films.

The Raman spectra of the AlScN/${\mathrm{Al}}_{2}$$O_{3}$ system, shown in Figure 3, are complex because of the multiple spectral bands of different origins. In order to properly assign them, the spectra have to be approached by inspecting the film and the substrate separately. The spectrum of the aluminium oxide substrate indicates the single-crystalline c-plane oriented corundum by the numerous sharp peaks. The most pronounced ones are at about 416.7, 576.3, and 748.8 ${\mathrm{cm}}^{-1}$, corresponding to the $A_{1g}$ and 2 $E_{g}$ modes, respectively [23]. These spectral bands are present in all Raman spectra shown, indicating the penetration depth of the laserline, which spanned through the entire depth of a nitride film. The spectrum of pure AlN exhibits the first-order Raman-active modes, such as $E_{2}$(low), $E_{2}$(high), and quasi $A_{1}$(LO) (labelled as “QLO”) [33]; and the second-order modes—optic overtone [$K_{3}$, M] and $A_{1}$-symmetry optic combination and overtone [34]. The spectral band at about 1189.4 ${\mathrm{cm}}^{-1}$ is assigned to the overtone of the $A_{1}$(TO) mode. The peak parameters, such as the spectral position and linewidth, are given in Table 1, to which we will refer from here on. According to the symmetry selection rules for the wurtzite-type crystals [35], the present combination of the visible spectral bands confirms the c-axis texture of the nitride films.

The spectral position of the $E_{2}$(high) mode in the spectrum of pure AlN (cf. Table 1) suggests a significant level of the tensile residual stress, which can be estimated to reach up to ca. 2 GPa, according to the Raman biaxial stress coefficient of −3.8 ${\mathrm{cm}}^{-1}$·${\mathrm{Gpa}}^{-1}$ and stress-free phonon frequency of 656.7 ${\mathrm{cm}}^{-1}$ [36]. Such a high value of the residual stress, beyond the yield strength of AlN (ca. 0.3 GPa), however, suggests that the biaxial stress is not the only contribution to the peak position. Moreover, wafer bow measurements revealed the residual stress of ca. 1.1 GPa [37]. As we show below, another important factor is the hydrostatic stress caused by diverse point defects. This is corroborated by the large linewidth values of all AlN bands, being inversely proportional to the phonon lifetime limited by phonon scattering. The phonons can be scattered by other quasiparticles, such as phonons and electrons, or the lattice irregularities. Considering the columnar microsctructures of the films, we expect significant contributions to the phonon scattering in AlN by the grain boundaries and overall point defects.

The Raman spectra of the AlScN films evolve with the Sc concentration (Figure 3) in perfect agreement with the previous reports [12,15]. Namely, the peaks, which correspond to the $E_{2}$(high) and QLO bands, shift towards lower frequencies, making the trend inversely proportional to the Sc concentration. We also observed a drastic enhancement of the bands above 900 ${\mathrm{cm}}^{-1}$, corresponding to the two-phonon modes, which may be related to the resonance enhancement in AlScN, the dielectric function of which drastically changes upon alloying [9]. Moreover, the expected high defect density promotes the midgap electronic states. The defect-state electron transitions in the visible light range can thus facilitate the resonance enhancement of the Raman scattering effect [38]. Apart from the three modes observed in the spectrum of the AlN film, two more bands at around 963.5 and 1071.8 ${\mathrm{cm}}^{-1}$ are evident for AlScN. Their assignment to the combination of the acoustic and optic modes, for example, TA + $A_{1}$(TO) or $E_{2}$(high), requires further investigations. The assignment of the two-phonon modes to the “interference fringes” [15] can be ruled out by the fact that the peak positions (cf. Table 1) are also redshifted, though not as drastically as in the case of the first-order modes. Another observation concerns the overall amplification of the spectral background, which may be attributed to the relaxation of the Raman selection rules as a result of the lattice disorder, enabling the visibility of the phonon density of states (pDOS). This is the reason for the absence of the $E_{2}$(low) mode in the spectra of AlScN. The relaxation of the momentum (*q*) conservation, observed as the enhancement of the background signal and typical broadening of the spectral features, is well known from the studies of other III-V solid solutions [33,39,40,41,42]. The origin of the relaxation is the substitutional disorder, which leads to the activation of the non-Γ-point (q≠0) phonon modes [41], which may either be seen as separate peaks or as peak tails contributing to the asymmetry of the pristine first-order modes [40]. Their actual spectral appearance is governed by the curvature of the phonon dispersion relations in the Brilloun zone. Given that AlScN pseudobinary alloys represent the amalgamation type of optical phonon behaviour [42], and since rock-salt ScN exhibits no first-order modes, no localisation of disorder structure is expected [41]. This implies the typical effects of the compositional fluctuation [43], and thus the asymmetric peak broadening and the non-linear linewidth variation with the Sc concentration, which is obvious from the retrieved peak characteristics (cf. Table 1). Due to the absent phonon dispersions for AlScN, we can approximate the behaviour of spectral modes using the ones of AlN [33,34], focusing on the two main first-order modes, $E_{2}$(high) and QLO, visible in all spectra (Figure 3). One can clearly see that the dispersions of these two modes are dramatically different: while the $E_{2}$(high) mode almost does not disperse from the zone-centre towards the edges, the QLO one spreads in the range of 200 ${\mathrm{cm}}^{-1}$ in Γ-H direction. This difference explains the stark asymmetry of the QLO mode in the spectra of AlScN, while $E_{2}$(high) remains symmetric even at the highest Sc concentration. The further theoretical studies would be necessary to verify this likely interpretation. We can hence conclude that the disorder-activated spectral features can be understood in the framework of typical compositional disorder, which originates from alloying, and the high-degree long-range ordering in the films is validated by the corresponding XRD patterns.

Thus, the evolution of the Raman bands due to Sc alloying can be traced only for two one-phonon bands and two-phonon bands (observed in AlN). Using the knowledge of the residual film stress in the AlScN films [37], we can estimate the shift of $E_{2}$(high) mode as a function of Sc concentration, *x*, assuming that the Raman biaxial stress coefficient is invariable:

Δω=−126.14·x

The slope factor of −126.14 is obtained via a linear fit of the peak position values (cf. Table 1) with a subtracted contribution from the residual film stress. Comparing the slope to the one reported by Deng et al. [12], our slope factor is almost two times lower, which stems from the fact the Raman peak is visible at much higher Sc concentrations, confirming the high quality of these epitaxial films. Additionally, we surmise that the biaxial stress coefficient of the $E_{2}$(high) mode in AlScN films may drastically differ from the one of AlN, which further increases the uncertainty for the application of Raman spectra for the Sc concentration determination.

**Figure 4:**
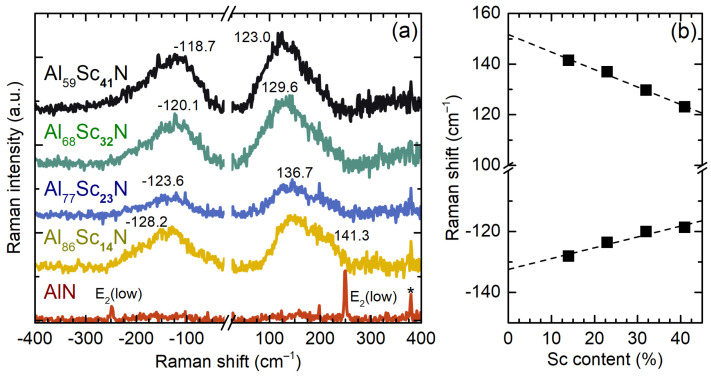
(**a**) Raman spectra of AlScN films at the low frequency spectral region. The spectra are stacked for clarity. The spectral bands are labelled by the positions of their maxima. The asterisk marks the band related to the ${\mathrm{Al}}_{2}$$O_{3}$ substrate. (**b**) The positions of the low-frequency bands plotted as a function of the Sc concentration. The dashed lines are the linear fits.

Apart from the one-phonon Raman bands, the Raman spectra of AlScN films also feature bands in the low-frequency region (Figure 4a). The suppression of the intense Rayleigh peak via the notch filter also enables us to observe the anti-Stokes side of the spectra. In the spectrum of AlN, we can clearly observe the $E_{2}$(low) mode at 249.5 ${\mathrm{cm}}^{-1}$ (on the Stokes side) and its anti-Stokes counterpart at −249.5 ${\mathrm{cm}}^{-1}$. The intensity of the Stokes-side Raman band is substantially higher than the one of the anti-Stokes counterpart, in accordance with the Bose–Einstein distribution of phonons [44]. The alloying of AlN with Sc leads to the emergence of the intense and broad band between 100 and 250 ${\mathrm{cm}}^{-1}$ in the spectra of AlScN films. Moreover, its spectral position is seemingly proportional to the Sc content (Figure 4b). The redshifted Stokes and anti-Stokes maxima and the peak intensities differ negligibly. Although their relation to Sc alloying is clear, the underlying mechanism of their Raman activity can only be controversially discussed. As discussed earlier, the alloying practically increases the overall defect density, which might lead to amorphisation in its extreme case. The latter is known to relax the momentum conservation law of the photon–phonon scattering process, allowing the detection of the phonon density of state in Raman spectra [45,46]. According to the phonon structure calculated for pure AlN [34] and ScN [47] lattices, no considerable density is expected to be observed in the spectral range below 200 ${\mathrm{cm}}^{-1}$. This fact rules out the assignment of the bands to the pDOS and a possible resonant enhancement of the acoustic phonons by the bandgap narrowing or alloy-induced absorption in AlScN. Surmising no relation to a particular crystalline phase, their origin may have a purely geometry nature. This is corroborated by the similar peak intensity, which contradicts the ratio of the intensity values of the Stokes and anti-Stokes bands dictated by the Bose–Einstein statistics. The similar low-frequency bands were observed in the Raman spectra of various semiconductor nanoparticles [48,49] emerging due to the confinement of the spheroidal acoustic phonons [50,51]. The obvious linear dependence of the peak position on the Sc content (Figure 4b) resembles the $\omega_{max}\propto d^{-1}$ relation, where *d* is a particle diameter [48] suggesting that alloying AlN with more Sc leads to an increase in the particles’ average size. The existence of AlN nanoparticles can also be ruled out by the comparably large crystallites detected via the XRD study. Thus, the actual origin of the nanoparticles can only be assumed to stem from the rock-salt ScN phase. In addition, it is more energetically favourable for rock-salt phases to form the spherical nanoparticles due to the isotropy of their crystal structure.

While the fundamental softening mechanism of the one-phonon bands due to Sc alloying is understood [5,12], the means of their broadening are not sufficiently explained in the discussion of the phonon lifetime reduction [17]. From Table 1, it is clear that broadening of the one-phonon bands depends on the Sc concentration more then that of the two-phonon bands, which may be expected taking into account their probabilistic origin. We thus focus on the linewdiths, Γ, of the $E_{2}$(high) and QLO modes, for which the correlation length (i.e., mean free path) can be estimated as follows

$$l=\frac{s}{\sqrt{\Gamma \omega_{0}}}$$

where *s* is the dispersion parameter, having the same order as the acoustic velocity and $\omega_{0}$ is the mode position [52]. The longitudinal acoustic velocity was estimated via the relation between the elastic constant, $c_{33}$, and the film density, ρ [53], using the data obtained via the DFT simulation of AlScN pseudobinary alloys [54]:

$$V_{L}=\sqrt{\frac{c_{33}}{\rho }}$$

The theoretically obtained values of the film density agree well with the experimental ones, corroborating their use in our analysis [55]. The derived values of the acoustic velocity and the phonon correlation length can eventually be used to assess the point defect density, $l^{-3}$, provided in Table 2.

**Figure 5:**
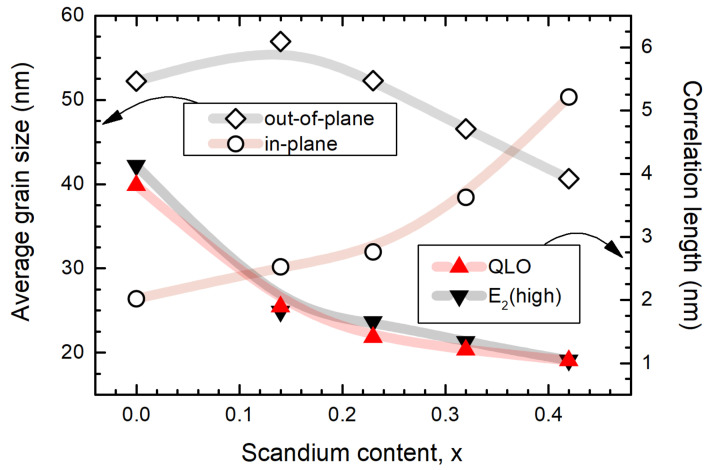
Average grain size and phonon correlation length as a function of the Sc content in the AlScN films. The continuous lines are given as guides to the eye.

The defect density value is two orders of magnitude larger in the case of AlScN with *x* = 0.42 of Sc when compared to pure AlN, which underlines the contribution of the point defects in the phonon scattering. This means that every 100th lattice position can present a certain defect, which, however, is still way too low to account for all substituting Sc atoms, comprising almost one half of Al atoms in the lattice of the sample with the highest Sc concentration. This example shows a very high sensitivity of the Raman scattering process, whereby even a minute presence of defects is reflected by the width of Raman peaks. Despite our assumption of the point defects due to alloying [56], another contribution to the defect density can be considered originating from the grain boundaries. The contributions from the phonon–phonon and phonon–electron scattering are discussed in the next section. To estimate the share of the scattering on grain boundaries, it is compelling to compare the phonon coherence length values to the average grain size in the AlScN films. The average size of the crystallites (i.e., coherently diffracting crystalline domains) can be estimated using XRD symmetric scans (Figure 1) via the well-known Scherrer Equation [57] assuming the shape factor of 0.9. We employed the AlScN (0002) reflection accessing the crystallite size in the out-of-plane direction, i.e., perpendicular to the film surface. Provided the anisotropy of the film growth due to the columnar structure of the nitride films, the in-plane grain size can be retrieved from the AFM topography images via the grain analysis algorithms. The average grain size values are plotted together with the phonon coherence lengths as a function of the Sc concentration (Figure 5).

The out-of-plane grain size of AlN grains was determined to be twice as large compared to the in-plane size, which agrees with the c-axis texture and the columnar growth of the nitride. The difference in the grain size values becomes smaller with the higher Sc content, reaching the equal magnitudes for the samples with *x* > 0.3 due to the larger grains seen via AFM (Figure 2) and the reduction of the grains in the orthogonal direction. The decrease in the out-of-plane grain dimension was observed by a similar trend in the phonon correlation length values, determined for the $E_{2}$(high) and QLO modes separately. Despite the different polarisation of the Raman modes, the phonon correlation length of the double-degenerate $E_{2}$(high) mode follows the one of the out-of-plane QLO modes, suggesting the phonons also travel less along the basal plane of the AlScN lattice. Even though this interpretation contradicts the positive trend of the in-plane size growth shown in Figure 5, the discrepancy may arise due to the worm-like surface morphology facilitated by clustering of individual grains. Thus, the similar trends in the correlation length values estimated using both Raman modes indicated the isotropic distribution of the scattering centres, suggesting point defects to be responsible for the low correlation length values. The phonon correlation length was estimated to be one order of magnitude lower than the average grain size in the out-of-plane direction overall, indicating that the lattice irregularities are ubiquitous and they become even more pronounced with the addition of Sc. We can hence speculate that the grain boundaries play a lesser role in the scattering of the optical phonons. Provided that the point defects also contribute to the hydrostatic stress in the lattice, high point defect density influences the spectral position of the mode itself, which explains the “impossibly high” residual biaxial stress in the AlN sample.

### 3.3. Temperature-Dependent Raman Measurements

**Figure 6:**
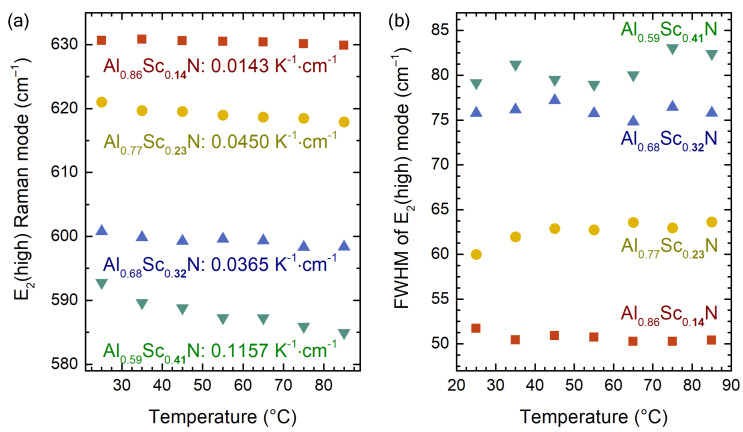
(**a**) $E_{2}$(high) mode position and (**b**) FWHM of AlScN films with various Sc concentration plotted as a function of temperature. The values given in (**a**) are the Raman temperature coefficients determined from the linear fit of the data.

Figure 6 shows the position and the linewidth of the $E_{2}$(high) mode obtained from the Raman spectra recorded at the elevated temperatures. The softening of the mode with the film heating was observed for all the AlScN films with different Sc contents (Figure 6a), which indicates the thermal expansion of the lattice due to the increase in the anharmonic phonon–phonon interactions, as expected. The phonon softening in this narrow temperature range is linear [58], conveniently enabling the temperature monitoring of the thin films. The linear approximations of the temperature coefficient [58] for the $E_{2}$(high) mode, shown in Figure 6a, varied with the Sc content. Compared to the one in AlN (0.0222 $K^{-1}\cdot$${\mathrm{cm}}^{-1}$ [59]), the increase in the coefficient values can be seen reaching up to 0.1157 $K^{-1}\cdot$${\mathrm{cm}}^{-1}$ for the highest Sc content. The five-fold difference suggests the dramatic changes in the anharmonic potential in AlScN corresponding to the stronger interatomic interaction [60], which contradicts the widely observed softening of the one-phonon bands as a result of bond length increase [12,15]. Additionally, the softening of the material should be accompanied by the lowering of the coefficient of the thermal expansion (CTE), but the experimental results showed the opposite behaviour [20]. The mechanism behind the ascending temperature coefficient should be related to the increase in the bound charge carrier density, realised through the alloying with Sc and the defect formation. It is instructive to see that the film with the least Sc added resulted in a temperature coefficient value (0.0143 $K^{-1}\cdot$${\mathrm{cm}}^{-1}$) lower than the one in AlN. The similarly low temperature coefficient values were found for CVD-grown AlScN with 20% of Sc [19]. Thus, we can conclude that the temperature coefficient is not only a function of the Sc concentration, but it also depends on the defect density, which may significantly alter the temperature monitoring.

In contrast to the clear trends in the peak position, the peak linewidth (FWHM), also known as the damping constant, weakly increases with the temperature increase (Figure 6b). Moreover, it may seem inconsistent between the AlScN films with different Sc concentrations. Despite the large peak widths, the determination error increases proportionally to the alloying degree owing to the possible contributions from the enhanced signals of the pDOS. Assuming a considerable uncertainty in the FWHM values, we conclude that the damping constant values failed to show any temperature dependence, and the fluctuation of the values was caused by a fitting uncertainty exclusively. This interpretation leads to the conclusion that the low phonon lifetimes as a result of the considerable point defect density cloak the anharmonic phonon–phonon scattering contribution usually responsible for the temperature-related peak broadening [61].

## 4. Conclusions

The vibrational structure of AlScN pseudobinary alloys in the thin-film form with various Sc content grown on sapphire substrates was investigated in this work by means of Raman spectroscopy. The films were confirmed to exhibit the wurtzite structure with the c-axis orientation facilitated by the columnar growth and the pebble-like surface, regardless of Sc content. Apart from the modes expected for the AlN-based wurtzite lattice, the Raman spectra of the AlScN films exhibited an enhanced phonon density of states and the long-time neglected second-order phonon modes. The one-phonon modes were used to analyse the defect density, which rose by almost two orders of magnitude in the case of AlScN with the highest Sc content examined. The comparison between the phonon correlation length and the average grain size showed the dominant role of the point defects originating largely due to the alloying. The temperature-dependent Raman measurements enabled the temperature coefficients for the $E_{2}$(high) mode to be determined, which can be used for the temperature monitoring in the AlScN-based devices. Using the near-excitation tunable notch filter, the low-frequency Raman spectra were recorded for the first time, demonstrating the Raman-active bands between 100 and 150 ${\mathrm{CM}}^{-1}$ attributed to the acoustic phonons confined in the spherical nanoparticles. We showed that Raman spectroscopy allows multifaceted material characterisation in the field of III-V compounds and their alloys, owing to the robust vibrational properties of the condensed matter. This work enables further studies of the vibrational properties of ${\mathrm{Al}}_{1-x}$${\mathrm{Sc}}_{x}$N alloys concerning the dependence of pressure and temperature in broader ranges.

## Figures and Tables

**Table 1:** Peak position and linewdith (FWHM) of the Raman-active bands collected from the AlScN films with the various Sc concentrations.

	$E_{2}$(high)	QLO	(O + A)${}_{1}$	(O + A)${}_{2}$	2$A_{1}$(TO)	Overtone [$K_{3}$, M]	$A_{1}$ Optic Comb & over
	Position (FWHM) [${\mathrm{cm}}^{-1}$]	Position (FWHM) [${\mathrm{cm}}^{-1}$]	Position (FWHM) [${\mathrm{cm}}^{-1}$]	Position (FWHM) [${\mathrm{cm}}^{-1}$]	Position (FWHM) [${\mathrm{cm}}^{-1}$]	Position (FWHM) [${\mathrm{cm}}^{-1}$]	Position (FWHM) [${\mathrm{cm}}^{-1}$]
AlN	649.1 (12)	883.7 (10)			1189.4 (16)	1258.1 (23)	1347.3 (35)
${\mathrm{Al}}_{0.86}$${\mathrm{Sc}}_{0.14}$N	630.2 (53)	852.1 (36)	963.5 (38)	1071.8 (77)	1185.6 (57)	1241.8 (45)	1303.5 (79)
${\mathrm{Al}}_{0.73}$${\mathrm{Sc}}_{0.23}$N	622.6 (55)	839.0 (57)	952.9 (85)	1061.6 (87)	1169.9 (55)	1232.0 (47)	1292.3 (94)
${\mathrm{Al}}_{0.68}$${\mathrm{Sc}}_{0.32}$N	603.8 (72)	824.5 (65)	942.0 (123)	1054.3 (89)	1156.4 (53)	1221.3 (52)	1282.6 (84)
${\mathrm{Al}}_{0.59}$${\mathrm{Sc}}_{0.41}$N	592.2 (94)	814.6 (69)	930.0 (147)	1052.3 (85)	1149.5 (54)	1215.9 (53)	1279.0 (95)

**Table 2:** Material properties of the AlScN pseudobinary alloys, such as the elastic constant, $c_{33}$, and the mass density, ρ [54] necessary to estimate the longitudinal sound velocity, $V_{l}$, and the point defect density, ${\left(l\right)}^{-3}$. The mode position and linewidth are taken from Table 1.

	$c_{33}$ [GPa]	ρ [g·${\mathrm{cm}}^{-3}$]	$V_{l}$ [m $s^{-1}$]	${\left(l\right)}^{-3}$ [ ${\mathrm{cm}}^{-3}$]
AlN	351.7	3.194	10,493.5	1.65 × 10${}^{19}$
${\mathrm{Al}}_{0.86}$${\mathrm{Sc}}_{0.14}$N	292.8	3.240	9506.0	1.91 × $10^{20}$
${\mathrm{Al}}_{0.73}$${\mathrm{Sc}}_{0.23}$N	253.1	3.268	8800.2	2.54 × $10^{20}$
${\mathrm{Al}}_{0.68}$${\mathrm{Sc}}_{0.32}$N	211.9	3.295	8020.4	4.73 × $10^{20}$
${\mathrm{Al}}_{0.59}$${\mathrm{Sc}}_{0.41}$N	169.3	3.319	7141.7	9.75 × $10^{20}$

## Data Availability

The data that support the findings of this study are available from the corresponding author upon reasonable request.
